# Decline of influenza-specific CD8^+ ^T cell repertoire in healthy geriatric donors

**DOI:** 10.1186/1742-4933-8-6

**Published:** 2011-08-16

**Authors:** Jessica B Lee, Mathias Oelke, Lakshmi Ramachandra, David H Canaday, Jonathan P Schneck

**Affiliations:** 1 Department of Pathology, Johns Hopkins University, 733 N Broadway BRB 632, Baltimore, MD, 21205, USA; 2 Department of Pathology, Case Western Reserve University, Wolstein 6530, 2103 Cornell Rd, Cleveland, OH, 44106, USA; 3 Scientific Review Program, Division of Extramural Activities, National Institute of Allergy and Infectious Diseases, National Institutes of Health, Bethesda, MD, 20892, USA; 4 Geriatric Research, Education and Clinical Center, Cleveland VA, 10701 East Blvd., Cleveland, OH, 44106, USA

## Abstract

**Background:**

While influenza vaccination results in protective antibodies against primary infections, clearance of infection is primarily mediated through CD8^+ ^T cells. Studying the CD8^+ ^T cell response to influenza epitopes is crucial in understanding the disease associated morbidity and mortality especially in at risk populations such as the elderly. We compared the CD8^+ ^T cell response to immunodominant and subdominant influenza epitopes in HLA-A2^+ ^control, adult donors, aged 21-42, and in geriatric donors, aged 65 and older.

**Results:**

We used a novel artificial Antigen Presenting Cell (aAPC) based stimulation assay to reveal responses that could not be detected by enzyme-linked immunosorbent spot (ELISpot). 14 younger control donors and 12 geriatric donors were enrolled in this study. The mean number of influenza-specific subdominant epitopes per control donor detected by ELISpot was only 1.4 while the mean detected by aAPC assay was 3.3 (p = 0.0096). Using the aAPC assay, 92% of the control donors responded to at least one subdominant epitopes, while 71% of control donors responded to more than one subdominant influenza-specific response. 66% of geriatric donors lacked a subdominant influenza-specific response and 33% of geriatric donors responded to only 1 subdominant epitope. The difference in subdominant response between age groups is statistically significant (p = 0.0003).

**Conclusion:**

Geriatric donors lacked the broad, multi-specific response to subdominant epitopes seen in the control donors. Thus, we conclude that aging leads to a decrease in the subdominant influenza-specific CTL responses which may contribute to the increased morbidity and mortality in older individuals.

## Background

In the United States, it is estimated that more than 30,000 people die each year as a result of influenza infection with over 90% of deaths in individuals over age 65 [1,2]. This is due, in part, to the diminished immune response in the elderly [3-7]. While antibodies protect against development of primary influenza infection, clearance of the infection is chiefly mediated through CD8^+ ^T cells [8,9]. It has been shown that CD8^+ ^T cells are protective against influenza infection and are critical for the clearance of influenza infection in animal models [10-15]. Thus, it is necessary to study host CD8^+ ^T cell response to influenza epitopes for a better understanding of susceptibility and changes that occur with aging.

In influenza, the HLA-A2 restricted response to the matrix protein peptide, M1_58-66_, is considered to be immunodominant [16-18]. However, recent studies of influenza have also shown a wide array of other epitopes, indicating that infection with influenza A induces a broader response [16,19-21]. Based on those studies [21], an alternative definition has been proposed of the hierarchy of dominant and subdominant epitopes for human immune responses based on the frequency and magnitude of response [22].

To assess the breadth and depth of influenza-specific immune responses, we compared enzyme-linked immunosorbent spot (ELISpot) analysis to a novel artificial Antigen Presenting Cells (aAPC) based stimulation. We found that the aAPC based stimulation assay was a more sensitive method to detect the breadth of influenza-specific responses. Using the aAPC assay to stimulate influenza-specific CD8^+ ^T cells *ex vivo *from younger control donors, aged 21-42, and geriatric donors, over the age of 65, we found responses against the immunodominant influenza M1_58-66 _peptide in both control and geriatric groups. Responses generated against the subdominant peptides, PB1_413-421_, NS1_123-132_, NA_231-239_, NA_75-84_, PA_46-54_, and PA_225-233 _were primarily seen in the control group. In contrast, the geriatric donors lacked the broad, multi-specific response to the subdominant influenza epitopes. These results indicate that aging leads to a narrowed influenza-specific subdominant memory CD8^+ ^T cell repertoire.

## Results

### Precursor frequency of influenza-specific cells

**Figure 1:**
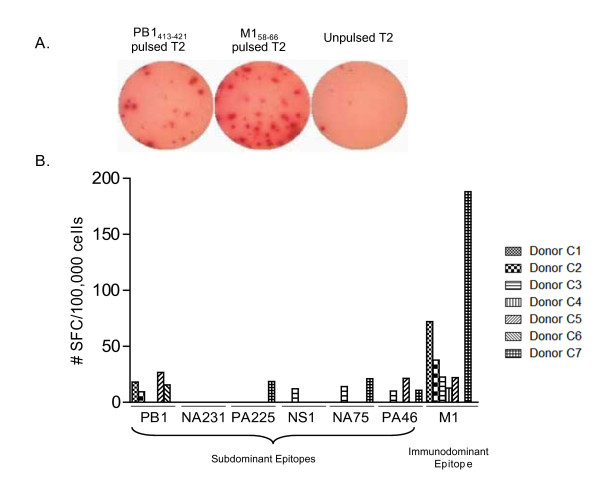
**Precursor frequencies of subdominant influenza-specific CD8^+ ^T cells by ELISpot**. CD8^+ ^T cells were obtained from control donor PBMCs and analyzed by ELISpot directly *ex vivo*. A. Shows a representative example of ELISpot wells. T2 cells were pulsed with either PB1_413-421 _peptide, M1_58-66 _peptide, or no peptide. CD8^+ ^T cells were plated at a 1:1 ratio with peptide pulsed T2 cells. B. Summary of IFNg secreting CD8^+ ^T cells determined using an ELISpot assay. The results are an average mean of triplicates with background subtracted. All displayed data points are statistically significant (p < 0.05), with a cut off value of 5 SFC/100000 CD8^+ ^T cells.

The precursor frequency of influenza-specific T cells was determined by an IFNg ELISpot assay on PBMC directly *ex vivo*. We analyzed the response to HLA-A2 restricted immunodominant and six subdominant influenza-specific peptides (Table 1) in seven of the control donors, aged 21-42 (Table 2). Few donors had detectable CD8^+ ^T cell precursor levels to multiple influenza-specific subdominant epitopes (Figure 1). Only four out of seven donors showed a significant (p < 0.05) response to PB1_413-421_, while three donors responded to PA_46-54 _and two donors to NA_75-84. _One donor responded to NS1_123-132 _or PA_225-233_, and no donors responded to NA_231-239 _(Figure 1B). Based on this and Gianfrani et al.'s study [21], we initially estimated a limited subdominant repertoire in normal control donors.

**Table 1:** Influenza peptides separated by pool

Peptide name	Peptide Sequence
Subdominant Peptides Pool 1	
PB1_413-421 _^a, d^	NMLSTVLGV
NA_231-239 _^b^	CVNGSCFTV
PA_225-233 _^a, e^	SLENFRAYV
Subdominant Peptides Pool 2	
NS1_123-132 _^c^	IMDKNIILKA
NA_75-84 _^a, e^	SLCPIRGWAI
PA_46-54_^a, d^	FMYSDFHFI
Immunodominant	
M1 _58-66 _^d^	GILGFVFTL

Influenza peptides separated into pools

Source- ^a ^Peptides were selected from Gianfrani et al. [21], ^b ^Peptides were selected from Daly et al.[40], ^c ^Peptides were selected from Kasprowicz et al. [41].

^d ^Indicates peptides were found to be completely conserved in all strains of influenza, Gianfrani et al. [21].

^e ^Indicates peptides were found to have one conservative amino acid substitution in 95% or more of the influenza strains tested, Gianfrani et al. [21].

**Table 2:** Demographic characteristics of Control Donors

Donor	Age	Sex	Last Flu vaccination (years prior)
C1	25	M	Never
C2	27	F	1
C3	25	M	Never
C4	42	M	2
C5	34	F	1
C6	31	M	2
C7	30	M	2
C8	30	M	1
C9	35	M	1
C10	32	M	> 5
C11	26	M	1
C12	32	M	1
C13	30	M	1
C14	30	F	5

Demographic characteristics of Control Donors

### Stimulation of subdominant influenza-specific CD8^+ ^T cells using aAPC

Since the precursor frequencies for the subdominant CD8^+ ^T cell specific response may be below the level of detection by ELISpot, we compared the ELISpot assay to an aAPC based stimulation assay initially developed for stimulation of viral CMV-immunodominant antigen-specific cells [23]. Here, we tested if this approach would be useful in stimulating influenza subdominant-specific CD8^+ ^T cells. For these studies, we modified the protocol by combining individually peptide-pulsed aAPC into pools of preloaded peptide-pulsed aAPC and stimulated purified CD8^+ ^T cells with the pools of aAPC (Table 1). The pools of aAPC were plated at a 1:1 ratio to CD8^+ ^T cells. After 3 rounds of weekly stimulation, we analyzed our cultures by HLA-multimer staining and intracellular cytokine staining (ICS).

**Figure 2:**
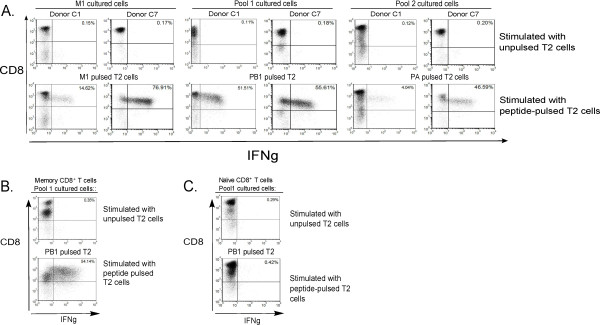
**IFNg secretion by antigen-specific immunodominant and subdominant CD8^+ ^T cells**. CD8^+ ^T cells were stimulated weekly for 3 weeks with M1_58-66_-aAPC (left panels), with pool 1 aAPC, which included NA_231-239_-aAPC, PA_225-233_-aAPC, and PB1_413-421_-aAPC (middle panels), or with pool 2 aAPC, which included NA_75-84_-aAPC, PA_46-54_-aAPC, and NS1_123-132_-aAPC (right panels). A) IFNg production by aAPC stimulated CD8*^+ ^*T cells from donor C1 and donor C7 after three rounds of stimulation as determined by ICS. CD8^+ ^T cells were stimulated with unpulsed T2 cells, M1_58-66 _pulsed T2 cells, PB1_413-421 _pulsed T2 cells, or PA_46-54 _pulsed T2 cells at a 1:1 ratio. B and C) Naïve CD8^+ ^T cells were separated and then stimulated weekly for three weeks with pool 1 aAPC. B) IFNg production by aAPC stimulated memory CD8^+ ^T cells from donor C1. CD8^+ ^T cells were stimulated with unpulsed T2 cells or PB1_413-421 _pulsed T2 cells. C) IFNg production by aAPC stimulated naïve CD8^+ ^T cells from donor C1. Naïve selected CD8^+ ^T cells were stimulated with unpulsed or PB1_413-421 _pulsed T2 cells.

Using aAPC based stimulation, we were able to generate peptide-specific CD8^+ ^T cells against the immunodominant, M1_58-66 _epitope, as well as the subdominant influenza-specific epitopes. Donor C1 and C7, representative examples, had approximately 15% and 77% M1_58-66_-positive CD8^+ ^T cells, respectively, based on IFNg ICS (Figure 2A, left panels). These cells were functional as the IFNg expressing population also coexpressed degranulation marker, CD107a (data not shown).

aAPC also stimulated expansion of the subdominant epitope-specific CD8^+ ^T cells. From the pool 1 aAPC cultures, 51% of Donor C1's and 56% of Donor C7's CD8^+ ^T cells were specific for PB1_413-421 _(Figure 2A, middle panels). Similarly, PA_46-54_-specific CD8^+ ^T cells were obtained using aAPC loaded with pool 2 peptides for both representative donors, C1 and C7 (Figure 2A, right panel). We did not find any donors that stained positive by multimer, but did not express IFNg by ICS (data not shown). Therefore, for standardization and comparison purposes, we analyzed all donors by ICS.

To determine if the aAPC based stimulation was expanding the memory CD8^+ ^T cell population or the naïve population, we isolated naïve CD8^+ ^T cells from control donors. After three rounds of stimulation, the aAPC expanded subdominant epitope specific cells from non-selected total CD8^+ ^T cells isolated from PBMCs (Figure 2B). However, the aAPC did not expand any subdominant epitope specific cells from the naïve-selected CD8^+ ^T cells (Figure 2C). Donor C1, a representative example, had approximately 54% PB1-positive CD8^+ ^T cells, based on IFNg ICS after 3 weeks of stimulation by pool 1 aAPC (Figure 2B). In contrast, no influenza-specific cells could be expanded from the naïve CD8^+ ^T cells (Figure 2C).

### Influenza-specific memory CD8^+ ^T cell response in younger donors is broad and multi-specific

**Figure 3:**
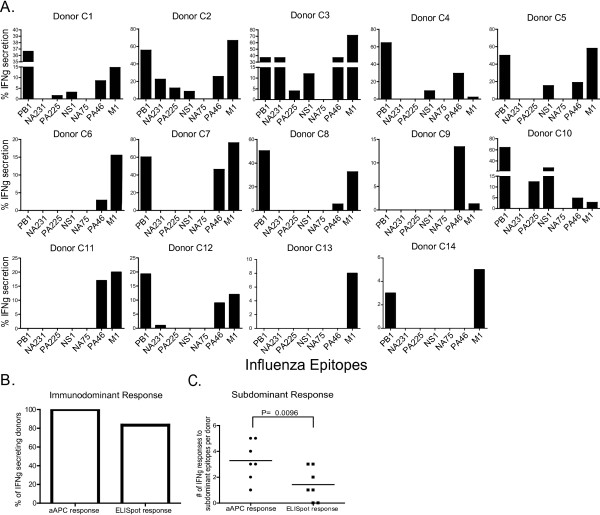
**Younger donors' CD8^+ ^T cell response to influenza A epitopes**. A) Frequency of peptide specific cells from control donors after three weeks of aAPC stimulation as determined by ICS for IFNg secretion and analyzed by flow cytometry. A positive result is defined as a clear population of cells secreting IFNg 5 fold above background. Background levels were determined by stimulating CD8^+ ^T cells with unpulsed T2 cells, as described in Figure 2. Figure 2 is a representative example of background levels for all donors. All data points displayed are 5 fold above background. B) Comparison of the immunodominant response in control donors. The percent of positive donors with peptide specific cells is compared to the percent of positive donors for IFNg secretion by ELISpot at wk 0 from control donors. C) Comparison of aAPC based stimulation to ELISpot assays for the subdominant influenza-specific responses.

The aAPC based stimulation revealed a broad CD8^+ ^T cell response to subdominant influenza-specific epitopes in the control donors (Figure 3A). Each donor has their own subdominant CD8^+ ^T cell response profile, and many donors responded to multiple subdominant epitopes. Several subdominant epitopes, PB1_413-421_, NS1_123-132_, and PA_46-54_, elicited a response from a majority of the donors. Furthermore, the magnitude of the response to the subdominant and immunodominant epitopes varied between each donor (Figure 3A).

Pools of peptide-loaded aAPC were able to stimulate multiple antigen-specific T cell populations simultaneously. Depending on the donor, 2 or 3 different subdominant CD8^+ ^T cell responses could be seen within each pool. For example, pool 1 stimulated three different antigen-specific CD8^+ ^T cells from Donors C2 and C3, and pool 2 stimulated two antigen-specific CD8^+ ^T cells from the same donors (Figure 3A).

aAPC based stimulation uniquely revealed responses not detected by ELISpot. Using single blood donations from a set of seven donors, we compared the sensitivity of the ELISpot assay to aAPC based stimulation assay. Both methods were comparable in detecting the immunodominant M1 specific responses; 100% of donors responded by aAPC stimulation and 83% by ELISpot (Figure 3B). However, the detection of subdominant specific T cells by aAPC expansion was significantly greater than their detection by ELISpot. The mean number of subdominant epitopes per donor detected by ELISpot was only 1.4 while the mean detected by aAPC assay was statistically higher at 3.3, p = 0.0096 (Figure 3C). Therefore, we based our repertoire comparison using the aAPC based stimulation assay.

### Influenza-specific CD8^+ ^T cell responses in geriatric donors

**Figure 4:**
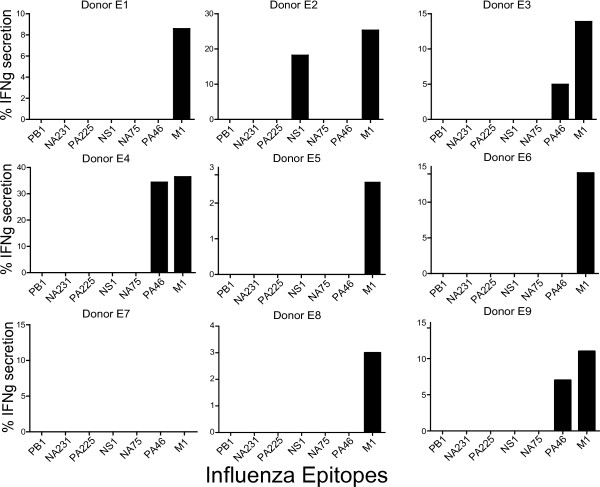
**Geriatric donors' CD8^+ ^T cell response to influenza A epitopes**. A) Frequency of peptide specific cells from geriatric donors after three weeks of aAPC stimulation as determined by ICS for IFNg secretion and analyzed by flow cytometry. Background levels were determined by stimulating CD8^+ ^T cells with unpulsed T2 cells, as described in figure 2. Figure 2 is a representative example of background levels for all donors. All data points displayed are 5 fold above background.

**Figure 5:**
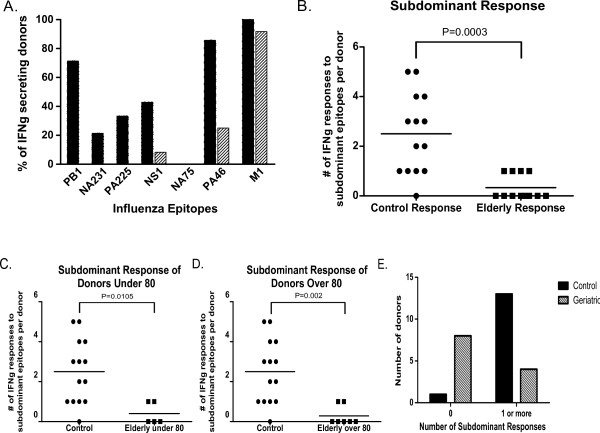
**CD8^+ ^T cell immune response to subdominant influenza epitopes in the younger and older populations**. A) Comparison of CD8^+ ^T cell response between control donors and geriatric donors based on IFNg secretion by ICS after three weeks of aAPC stimulation. Filled bars are control donors, lined bars are geriatric donors. B) Comparison of the number of responses to subdominant epitopes per donor, comparing control to geriatric donors. Positive responses were determined by ICS for IFNg secretion after three weeks of aAPC stimulation. C) Comparison of number of responses to subdominant epitopes per donor, comparing control to geriatric donors under the age of 80. D) Comparison of number of responses to subdominant epitopes per donor, comparing control to geriatrics over the age of 80. E) Comparison of responding donors to either no subdominant influenza-specific epitopes or 1 or more subdominant influenza-specific epitopes. This difference is statistically significant, p < 0.0026, by Fisher's exact test.

We determined the breadth of influenza-specific CD8^+ ^T cells in older geriatric adults, aged 65 and above (Table 3). 11 of the 12 geriatric donors had responses specific for the immunodominant M1_58-66 _epitope (Figure 4). In contrast, geriatric donors lacked responses to most of the subdominant influenza peptides seen in the control group (Figures 3A and 4). Of the donors who did elicit a subdominant response, the breadth of their response was limited (Figure 4). As noted earlier, in the control, younger group, donors responded to as many as five of the six subdominant epitopes (Figures 3A and 5A). The mean number of subdominant epitopes per donor detected in the geriatric group was only 0.33, compared to the mean of 2.5 detected for control donors, p = 0.0003 (Figure 5B). Similarly, we separated the geriatric donors into two groups, under 80 and 80 and older, and compared the number of subdominant responses per donor in each group to the control donors' subdominant response. Both groups were statistically different from the control group with p = 0.01 for under 80 and p = 0.002 for donors above 80 years old (Figures 5C and 5D).

**Table 3:** Demographic characteristics of Geriatric Donors

Donor	Age	Sex	Last Flu vaccination (years prior)
E1	83	M	2
E2	83	F	2
E3	68	M	1
E4	67	F	> 5
E5	72	M	1
E6	70	M	1
E7	86	M	> 3
E8	86	M	1
E9	83	M	1
E10	85	M	1
E11	87	M	1
E12	69	M	2

Demographic characteristics of Geriatric Donors

To further verify the difference in response to subdominant epitopes between the control and geriatric donors, we grouped the responses to subdominant epitopes into no responses or one or more subdominant responses. Geriatric donors lacked a subdominant response and this was determined to be statistically significant by Fisher's exact test, p = 0.0026 (Figure 5E). None of the geriatric donors had multi-specific subdominant responses. Of note, Donors E2, E3, E4, and E9 responded to the subdominant epitopes NS1 or PA46, epitopes that were highly prevalent in the control group but none responded to another prevalent subdominant epitope, PB1_413-421 _(Figures 3A, 4 and 5A). Thus, in contrast to the broad, multi-specific subdominant response seen in control donors, there was a substantially more restricted response in geriatric donors.

## Discussion

In contrast to the broad CD8^+ ^T cell response to subdominant influenza epitopes seen in younger donors, we found a restricted response in geriatric donors. Our work is the first to document losses in subdominant influenza-specific memory responses that occur with aging in humans. Our findings are consistent with published studies that report that geriatric individuals maintain immunodominant, M1_58-66 _specific, CD8^+ ^T cells [24-26], but extend that work significantly as we report that the lack of breadth of subdominant influenza-specific T cells in the geriatric population even to the highly prevalent subdominant epitopes like PB1_413-421_. In younger donors, we also found that the majority of the donors had T cells that were specific for multiple subdominant epitopes which was larger than predicted, Gianfrani et al. [21]. Our study shows a narrowing of the CD8^+ ^T cell repertoire associated with ageing.

aAPC based stimulation detected influenza-specific T cells that were below the limits of detection by ELISpot assays. Using this novel technique, we were able to significantly enhance our ability to probe the breadth and depth of the human CD8^+ ^T cell repertoire against influenza. Interestingly, the aAPC stimulation assay revealed that three of the six subdominant influenza epitopes, PB1_413-421_, NS1_123-132_, and PA_46-54_, induce a response in the many of the control donors. These responses are very low frequency events which were only effectively detected in the aAPC assay. It would be interesting to investigate why T cells specific for these epitopes are present at such low precursor frequencies, as their prevalence may implicate an important role in the CD8^+ ^T cell immune response to influenza.

Our aAPC based stimulation assay overcomes limitations associated with other assays. Precursor frequency analysis of antigen-specific cells can be performed by a variety of assays including ELISpot and tetramer staining. As with all assays, they are limited by the number of events being analyzed and the background activity associated with each assay. In the aAPC assay, CD8^+ ^T cells are repetitively stimulated to expand antigen specific T cells to a level that is detectable by multiple methods, such as tetramer staining, ICS or ELISpot. In the process of the repetitive stimulation we lose information on the exact precursor frequency, but the aAPC stimulation allowed detection of a wider breadth of antigen specific T cells than were previously detected. By using the pools of aAPC, as opposed to individually peptide-pulsed aAPC per stimulation, these experiments were logistically feasible with a modest, 40 cc, blood donation.

The use of only moderate amounts of blood becomes a central issue in the analysis of the at risk geriatric population. Within the geriatric population the amount of blood drawn is restricted and volunteers' consent for multiple blood draws is limited. Thus, by using a single, modest blood draw we were able to recruit and enroll more geriatric donors.

Age-associated T cell repertoire changes have been previously reported. The diminished immune response in geriatric individuals is often attributed to thymic involution, which leads to a reduction in the thymic output of naïve T cells [27]. However, since the aAPC based stimulation assay only expands the memory CD8^+ ^T cell population (Figure 2B and 2C), the lack of subdominant specific T cells in the geriatric group is likely due to alterations in the memory CD8^+ ^T cell population and not a result of naïve CD8^+ ^T cell loss. Additionally, the M1 proliferative response was seen in all but one of the geriatric donors. Therefore, intrinsically all donors' T cells were capable of proliferating. Thus, we concluded that the precursor frequency of the subdominant influenza-specific responses, not their ability to proliferate, is selectively diminished in the older population.

Changes in the functional T cell repertoire are also known to occur in cytomegalovirus (CMV)-specific T cell responses in the geriatric population. Over time individuals lose their CD8^+ ^T cell diversity and their immune response to CMV is narrowed [28-30]. CMV is a virus that persists latently in the body and the narrowing of the CD8^+ ^T cell response is believed to be due to the persistence of CMV antigen throughout life. In contrast, influenza is not a latent virus, however individuals may be vaccinated and/or infected with influenza multiple times during their lifetime. These multiple exposures may lead to a shift in the T cell repertoire and a narrowing of the immune response that we observed in the geriatric population. Alternatively, the loss of the influenza-specific subdominant T cells in the geriatric donors might be a result of original antigenic sin. Original antigenic sin occurs when there are multiple infections with similar, but not identical viruses. The immune system is tricked into believing that the memory CD8^+ ^T cells produced in the initial infection are sufficient to ward off the infection with a similar virus and this leads to a narrowing in the immune response [31,32]. Lastly, narrowing of the CD8^+ ^T cell response may be attributed to heterologous immunity. Heterologous immunity occurs when memory CD8^+ ^T cells are activated during a secondary infection in response to a different virus [33-35]. It is possible that non-influenza viruses may induce cross-reactive responses to the immunodominant M1_58-66 _specific CD8^+ ^T cells, which leads to greater percentage of M1_58-66 _specific CD8^+ ^T cells, and a narrowing in the subdominant CD8^+ ^T cell immune response.

In contrast to the studies done in CMV, Boon et al. [36]studies indicate that the percentage of influenza-specific T cells does not change with age. In that work the authors looked at a global response to influenza by infecting PBMC directly *ex vivo *and looking for cytokine secretion by ICS [36]. They found no correlation with age and percentage of IFNg secreting CD8^+ ^T cells. However, they were unable to directly look at epitope specific responses. Based on the experimental design, we believe, that their analysis is likely focused on the immunodominant epitopes in both the young and older donors, since the precursor levels of subdominant-specific CD8^+ ^T cells are much lower, it is possible that the subdominant response is below their limits of detection. This highlights the importance of also studying the subdominant antigen-specific responses.

## Conclusions

Using the aAPC stimulation assay we were able to significantly enhance the ability to probe the depth of the human CD8^+ ^T cell repertoire against influenza in younger control and geriatric donors. We observed that all young donors had T cells specific for the immunodominant peptide, and that most had T cells that were specific for multiple subdominant epitopes as well. Compared to the broad responses seen in the younger control donors, there was a near total absence in the geriatric donors with maintenance of only the immunodominant M1_58-66_-specific response.

Our results have potential implication for vaccine design targeted at boosting influenza-specific CD8^+ ^T cells responses as it has been suggested that vaccine protection in geriatric donors correlates better with T cell responses than antibody responses [37]. Therefore, understanding the mechanism that leads to the loss of subdominant influenza-specific CD8^+ ^T cells may be crucial in designing a more effective vaccine for influenza and, more generally, for vaccines targeting the geriatric population.

## Methods

### Donors

All donors were HLA-A2^+ ^as typed by monoclonal antibody, BB7.2, or a PCR-based kit from (Biosynthesis). Donors in the control group consisted of both males and females, between the ages of 21-42, many of whom had previously received an influenza vaccine (Table 2). Geriatric donors were a mixed group of males and females, varied in the timing of their last influenza vaccine, and aged 67 and above (Table 3). The donors, control and geriatric, were healthy. The exclusion criteria were patients with cancer, immune disorders, or on immunosuppressant medication. All donor samples were obtained from Baltimore, MD, or Cleveland, OH, USA. Informed consent was obtained from all donors before enrolling in the study. The Institutional Review Boards of Johns Hopkins Medical Institutions, Case Western Reserve University, and the Cleveland VA approved this investigation.

### Peripheral blood mononuclear cells (PBMC)

Blood was obtained using VacutainerCPT or heparin green top tubes (Becton-Dickinson). PBMC were isolated by Ficoll-Hypaque (Amersham Pharmacia Biotek, Uppsala, Sweden) density gradient centrifugation. CD8^+ ^T cells were isolated from PBMC using the untouched human CD8^+ ^T cell isolation kit (Miltenyi). Naïve cells were further selected by secondary enrichment with naïve CD8^+ ^T cell isolation kit (Miltenyi).

### Cell lines

TAP (transporter associated with antigen processing)-deficient 174CEM.T2 (T2) cells were maintained in M' medium (RPMI 1640 medium (Gibco, Invitrogen Corporation), non-essential amino acids (Sigma-Aldrich), sodium pyruvate (Gibco, Invitrogen Corporation), vitamin solution (Gibco), 2-mercaptoethanol (Gibco), 10 μM ciprofloxacin (Serologicals Proteins Inc)) supplemented with 10% fetal calf serum (Atlanta Biologicals).

### Peptides

All peptides M1_58-66_: GILGFVFTL, PB1_413-421_: NMLSTVLGV, PA_225-233_: SLENFRAYV, NA_231-239_: CVNGSCFTV, PA_46-52_: FMYSDFHFI, NA_75-84_: SLCPIRGWAI, and NS1_125-132_: IMDKNIILKA were synthesized by GenScript (Table 1). Purity of all peptides (> 95%) was confirmed by mass-spectral analysis and high-pressure liquid chromatography.

### ELISpot assay

PVDF membrane-bottomed plates (Millipore) were coated with anti-IFNg antibody (EBiosciences). T2 cells were pulsed with 10 ug/mL peptide in serum-free M' media overnight at 37°C. CD8^+ ^T cells were isolated from PBMCs as described above. CD8^+ ^T cells and washed target cells were plated at a 1:1 ratio. Negative control wells contained unpulsed T2 cells with effector cells. The plates were incubated at 37°C for 16-20 hrs. The plates were washed with ELISpot wash buffer (PBS, 0.1% Tween-20) (Gibco, Sigma-Aldrich) and incubated first with secondary anti-IFNg mAb and then with avidin-HRP (EBiosciences) according to manufacturer's protocol. Plates were developed using AEC peroxidase substrate (Sigma-Aldrich). Colored spot-forming cells (SFC) were counted using an automated ELISpot reader (Immunospot, CellularTechnology). Each donor's peptide-pulsed stimulated wells were compared to their own unstimulated control well.

### Generation of artificial antigen presenting cells

A2-Ig based aAPC was prepared according to the previously described method [23]. A2-Ig molecules were loaded with 30 μg/ml of a single peptide (GenScript) in 1 ml PBS containing 5 × 10^7 ^beads and rotated overnight at 4°C. aAPC beads were stored in peptide solution at 4°C, with only a single peptide being loaded onto individual A2-Ig aAPC per vial. aAPCs were pulsed with the following peptides: M1_58-68 _(M1_58-68_-aAPC), NA_231-239_, PA_225-233_, PB1_413-421_, NA_75-84_, PA_46-54_, and NS1_123-132_.

### Expansion of primary human CD8^+ ^T cells

CD8^+ ^T cells (10^6^/plate) were co-cultured at a 1:1 ratio with peptide-loaded aAPC in a 96-well round-bottom plate (BD Falcon) with 165 μl/well M' medium, supplemented with 5% autologous plasma or 5% Human AB serum (HyClone) and 6% T-cell growth factor (TCGF) at 37°C in a 5% CO_2 _incubator. TCGF was prepared as previously described [38]. The culture media was replenished once a week on day 4. On day 7 CD8^+ ^T cells were harvested, counted and re-plated at a 1:1 ratio of CD8^+ ^T cells to fresh peptide-loaded aAPC. This was repeated weekly for up to 5 weeks. For the immunodominant M1_58-66 _generated CD8^+ ^T cells, cells were plated at a 1:1 ratio with only M1_58-68_-aAPC. For the subdominant epitopes, CD8^+ ^T cells were cultured at a 1:1 ratio of CD8^+ ^T cells to aAPC where the aAPC consisted of a pool of aAPC. Pool 1 consisted of NA_231-239_-aAPC, PA_225-233_-aAPC, and PB1_413-421_-aAPC. Pool 2 consisted of NA_75-84_-aAPC, PA_46-54_-aAPC, and NS1_123-132_-aAPC (Table 1). All aAPC were peptide loaded individually and then pooled when added to the plates with the CD8^+ ^T cells.

### Multimer staining and flow cytometric analysis

The antigen specificity was tested by staining with monoclonal antibody (clone UCHT-4, Sigma-Aldrich) and HLA-A2 tetramer PE loaded with either Mart-1 peptide (Mart-1 tetramer) for noncognate control, M1_58-66 _peptide (M1_58-66 _tetramer) (Beckman Coulter Inc., San Diego, CA), or A2-Ig dimer loaded with PB1_413-421 _peptide (PB1_413-421 _dimer), PA_225-233 _peptide, NA_231-239_, PA_46-52_, NA_75-84_, or NS1_125-132_. The noncognate dimer control was unloaded A2-Ig dimer. All A2-Ig dimer was prepared in our laboratory [39]. Samples were collected using a FACS Calibur flow cytometer with CELLquest software and were analyzed using FCS Express software.

### Intracellular cytokine staining and CD107a assay

aAPC (10^5^/well) generated CD8^+ ^T cells were placed in a single well of a 96-well flat-bottom plates (BD Falcon) at a 1:1 ratio with peptide pulsed or unpulsed T2 cells. Prior to stimulation, 10 μL anti-human CD107a PE-Cy5 (BD Pharmingen, San Diego, CA) were added to each well. After 1 hour of incubation GolgiStop (BD Pharmigen) was added to each well. Cells were incubated for up to 10 hours, then harvested, stained for with anti-CD8 APC (BD Pharmigen), fixed and permeabilized with CytoPerm/CytoFix (BD Pharmigen), and stained for cytokines with anti-IFNg FITC (BD Pharmigen) according to the manufacturer's protocol.

### Statistical Analysis

A paired T test was used to determine statistical significance in ELISpots. Pairwise analysis was performed when comparing the total number of subdominant responses per donor between control and geriatric donors using the Mann-Whitney test. Fisher's exact test was used to analyze the relationship between control and geriatric donors and their subdominant response. Statistical analysis was performed using a graphing and data analysis program (GraphPad Prism 5.01 for Windows, GraphPad Software, San Diego California USA, http://www.graphpad.com). Significance was defined as p < 0.05.

## Competing interests

The authors declare that they have no competing interests.

## Authors' contributions

JBL carried out the immunoassays, assay development, data analyses, statistical analyses, and drafted the manuscript. MO participated in the design of the study and helped with editing of the manuscript. LR participated in the data analysis and coordination of the study. DHC performed genotyping experiments of geriatric donors, participated in design and coordination of the study, and helped to draft the manuscript. JPS conceived of the study, and participated in its design and coordination, and helped to draft the manuscript. All authors read and approved the final manuscript.
